# Characteristics of Extended-Spectrum Beta-Lactamase-Producing Enterobacteriaceae and Contact to Animals in Estonia

**DOI:** 10.3390/microorganisms8081130

**Published:** 2020-07-27

**Authors:** Kaidi Telling, Age Brauer, Mailis Laht, Piret Kalmus, Karolin Toompere, Veljo Kisand, Matti Maimets, Maido Remm, Tanel Tenson, Irja Lutsar

**Affiliations:** 1 Department of Microbiology, Institute of Biomedicine and Translational Medicine, University of Tartu, 50411 Tartu, Estonia; irja.lutsar@ut.ee; 2 Institute of Molecular and Cell Biology, University of Tartu, 51010 Tartu, Estonia; age.brauer@ut.ee (A.B.); maido.remm@ut.ee (M.R.); 3 Institute of Technology, University of Tartu, 50411 Tartu, Estonia; mailis.laht@ut.ee (M.L.); veljo.kisand@ut.ee (V.K.); tanel.tenson@ut.ee (T.T.); 4 The Institute of Veterinary Medicine and Animal Sciences, Estonian University of Life Sciences, 51014 Tartu, Estonia; piret.kalmus@emu.ee; 5 Institute of Family Medicine and Public Health, University of Tartu, 50411 Tartu, Estonia; karolin.toompere@ut.ee; 6 Institute of Clinical Medicine, University of Tartu, 50406 Tartu, Estonia; matti.maimets@ut.ee; 7 Infection Control Department, Tartu University Hospital, 50406 Tartu, Estonia

**Keywords:** ESBL, *Escherichia coli*, healthy volunteer, one health, CTX-M, whole genome sequencing

## Abstract

We have attempted to define the prevalence and risk factors of extended-spectrum beta-lactamase-producing Enterobacteriaceae (ESBL-Enterobacteriaceae) carriage, and to characterize antimicrobial susceptibility, beta-lactamase genes, and major types of isolated strains in volunteers, with a specific focus on humans in contact with animals. Samples were collected from 207 volunteers (veterinarians, pig farmers, dog owners, etc.) and cultured on selective agar. Clonal relationships of the isolated ESBL-Enterobacteriaceae were determined by whole genome sequencing and multi-locus sequence typing. Beta-lactamases were detected using a homology search. Subjects filled in questionnaires analyzed by univariate and multiple logistic regression. Colonization with ESBL-Enterobacteriaceae was found in fecal samples of 14 individuals (6.8%; 95%CI: 3.75–11.09%). In multiple regression analysis, working as a pig farmer was a significant risk factor for ESBL-Enterobacteriaceae carriage (OR 4.8; 95%CI 1.2–19.1). The only species isolated was *Escherichia coli* that distributed into 11 sequence types. All ESBL-Enterobacteriaceae isolates were of CTX-M genotype, with the *bla*CTX-M-1 being the most prevalent and more common in pig farmers than in other groups. Despite the generally low prevalence of ESBL-Enterobacteriaceae in Estonia, the pig farmers may still pose a threat to transfer resistant microorganisms. The clinical relevance of predominant *bla*CTX-M-1 carrying *E. coli* is still unclear and needs further studies.

## 1. Introduction

The prevalence of extended-spectrum beta-lactamase-producing Enterobacteriaceae (ESBL-Enterobacteriaceae) has continuously increased in both hospitals and communities all over the world [1,2]. Its frequency varies widely (2–70%) and depends on geographical location [3,4,5]. These trends have been mainly associated with the spread of the highly pathogenic multi-resistant *Escherichia coli* ST131 carrying *bla*_CTX-M-15_ in healthcare institutions [6,7].

Traditionally, Estonia is regarded as region with a low antibiotic resistance similar to other Northern European countries. However, there is a trend showing that resistance rates of some enterobacterial species to 3rd generation cephalosporins have significantly increased over the last 10 years [8].

Mucosal colonization usually precedes invasive infection. Therefore, monitoring mucosal colonization provides an indication of antimicrobial resistance (AMR) rates in a country. A large variability in ESBL-Enterobacteriaceae colonization in Estonia′s neighboring countries, namely Latvia and Russia (1.6% vs. 23.3%, respectively) were found in a recent study by Ny et al. [3]. Thus, the importance of local knowledge is crucial when planning measures to tackle antibiotic resistance.

The carriage of ESBL-Enterobacteriaceae out-side the healthcare system has been associated with travel to high prevalence countries [9,10,11], as well as contact with pets or farm animals through occupation as food handlers or farmers. In recent years more and more data emerge on importance of cross species transmission of AMR [12,13]. It has been suggested that the digestive tract of livestock and poultry is a significant reservoir of multi-resistant bacteria. Thus, this may serve as a possible source of human colonization through either direct contact with animals or contaminated food chain and thus deserves further investigation [1,14,15].

Since 2000, Enterobacteriaceae producing CTX-M-ESBL has been predominant, with a wide genetic variability amongst humans outside the hospital, i.e., in the community. Certain geographical links have shown that *bla*_CTX-M-15_ is more prevalent in Europe, Africa, Middle East and South Asia, whereas *bla*_CTX-M-14_ is dominates in the Western Pacific Region [6,7,16]. In the Estonian clinical setting *bla*_CTX-M-15_ has also been dominating [17]. Unfortunately, data regarding beta-lactamase genes of ESBL-Enterobacteriaceae strains outside hospitals is still lacking.

A new successful *bla*_CTX-M_ type has emerged in recent years and has further confused the situation. Thus, *bla*_CTX-M-27_ was firstly discovered in France and has been considered as a single nucleotide variant of *bla*_CTX-M-14_ [18]. As *bla*_CTX-M-15_ and *bla*_CTX-M-14_ are mainly found in humans, *bla*_CTX-M-27_ and *bla*_CTX-M-1_ occur in many species, making cross-species transmission likely and transmission between different environments possible [14].

We hypothesized that colonization by ESBL-Enterobacteriaceae is greater in people in contact with farm animals or pets than in general population and that this carriage may be associated with human disease. Thus we aimed (i) to evaluate the extent of ESBL-Enterobacteriaceae carriage in otherwise healthy people, with a particular focus on those who are in contact with animals, (ii) analyze phenotypic antimicrobial resistance, beta-lactamase genes, and major subtypes of circulating strains, and (iii) attempted to characterize possible risk factors associated with acquisition of ESBL-Enterobacteriaceae.

## 2. Material and Methods

### 2.1. Study Design

This prospective study was a part of the research project “Transfer routes of antibiotic resistance” in Estonia (ABRESIST). All procedures were carried out in accordance with the ethical standards of the Research Ethics Committee of the University of Tartu (approvals 213/T-11 and 241/M-16 23th of April 2012), and in keeping with the 1964 Helsinki declaration and its later amendments, at comparable ethical standards. Informed consent was obtained from all individual participants (or their guardians) included in the study.

From 30th of April 2012 to 11th December 2013 we approached four groups of people as follows. First, we contacted veterinarians via the official mailing lists of the Estonian Small Animal Veterinary Association and Estonian Veterinary Association; second, we contacted pig farms with more than 1000 animals to recruit pig farmers; third, we approached dog owners via a website of Animal Clinic of the Estonian University of Life Sciences and finally we recruited subjects who should represent Estonian community. For that patients admitted for elective orthopedic surgery or in pediatric wards of the Tartu University Hospital were contacted if they were older than six months, had no antimicrobial treatment or hospitalization in the previous three months and time since hospitalization was of <48 h. Three former groups were those with contact to animals and the fourth group (patients) represented the general population. Each subject was included once and only one person per a household was recruited.

After signing an informed consent all subjects provided samples and filled in a questionnaire about their demographics (age, gender) and factors possibly influencing ESBL-Enterobacteriaceae carriage (occupation, main source of food and drinking water, smoking, travel together with destination in previous year, eating outside the home, contact with pets or farm animals, and antibacterial treatment and hospitalization in the previous 12 months).

### 2.2. Sample Collection and Analysis

Nasal swabs and stool samples were either self-collected (dog owners and veterinarians) following detailed instructions or sampled by the trained personal (pig farmers and control group). Nasal specimens were taken using swabs with transport medium (Transystem, COPAN, Italy) and 2–3 g of feces was taken with specialized spoon and placed into a container (Aptaca, Canelli, Italy). All samples were immediately transported to the microbiology laboratory of the University of Tartu and stored at −80 °C for a maximum of 2 months. If quick transportation was not available, samples were stored at −20 °C for a maximum of 48 h.

Thawed nasal swabs were plated onto blood agar and incubated at 37 °C for 24–48 h in ambient air. All colonies with different morphology were isolated and identified using MALDI-TOF mass spectrometry (Bruker Daltonics, Bremen, Germany).

Defrosted fecal samples were plated on selective medium for isolation of ESBL-producing organisms (BrillianceTM ESBL Agar, Oxoid, Basingstoke, UK) and incubated at 37 °C for 24 h. Then two colonies per plate with morphology suggestive of *Escherichia coli* or the *Klebsiella, Enterobacteria, Serratia*, and *Citrobacter* group (KESC) were selected and confirmed at species level using MALDI-TOF mass spectrometry (Bruker Daltonics, Bremen, Germany).

### 2.3. Antimicrobial Susceptibility Testing

All isolated Enterobacteriaceae were tested for ESBL production by measuring ceftazidime, cefotaxime and cefepime minimal inhibitory concentrations (MIC) alone and in combination with clavulanic acid (Etest, bioMérieux, Marcy l’Etoile, France). The Etest was also used to test additional susceptibility of ESBL-positive strains to meropenem, piperacillin/tazobactam, gentamicin, amikacin, ciprofloxacin, TMP/SMX, fosfomycin, and tigecycline. The quality control strain routinely used was *E. coli* ATCC^®^ 25922™. EUCAST breakpoints and definitions were used to interpret susceptibility [19,20].

### 2.4. DNA Extraction

DNA of the isolated ESBL-*Enterobacteriaceae* strains was extracted from single colonies grown on blood-agar using a modified GuSCN-silica protocol [21]. Briefly, cells were transferred to a solution containing 570 µL TRIS-EDTA buffer (pH 7.6). Then, 30 µL 10% SDS with ~0.5 g zirconium beads (0.1 mm diameter) was added and the mixture processed for 5 min on bead beater (Minibead beater, Bio Spec Products, Bartlesville, DA, USA), followed by centrifugation at 10,000 rpm for 1 min. Lysis involved combining the supernatant with 900 µL lysis buffer L6 (5.25 M GuSCN, 100 mM Tris—HCl pH 6.4, 20 mM EDTA, 1.3% Triton X-100) and 40 µL custom-prepared silica suspension. The mixture was incubated for 5 min at room temperature before being centrifuged at 5000 rpm for 10 s. The supernatant was discarded, and the pellet washed with 1000 µL buffer L2 (5 M GuSCN) and 1000 µL 50% ethanol. The silica pellet was briefly dried, and the DNA eluted with ultra-pure water (milli-Q). The extracted DNA was stored at −20 °C until analysis.

### 2.5. Whole Genome Sequencing

Total bacterial DNA was quantified using a Qubit^®^ 2.0 Fluorometer (Invitrogen, Grand Island, NE, USA) and 2200 TapeStation (Agilent Technologies, Santa Clara, CA, USA). Ten ng sample DNA was processed with an Illumina Nextera XT sample preparation kit (Illumina, San Diego, CA, USA). The resulting DNA libraries were validated by qPCR using a Kapa Library Quantification Kit (Kapa Biosystems, Woburn, MA, USA) to optimize cluster generation.

Then, 23 ssDNA Nextera XT libraries originating from 23 different clones were pooled and sequenced in one rapid-output run of a HiSeq2500 (Illumina, San Diego, CA, USA), with paired-end 150-bp reads. Demultiplexing used CASAVA 1.8.2. (Illumina, San Diego, CA, USA), allowing one mismatch in the index reads.

### 2.6. Draft Assembly of Whole Genome Sequences (WGS), in Silico Multi-Locus Sequence Typing (MLST) and Phylogeny Analysis

All Illumina reads were assembled de novo using the SPAdes genome assembler (ver 3.5.0) together with the MismatchCorrector [22].

A BLAST-based tool from https://cge.cbs.dtu.dk/services/MLST/ was run to annotate the MLST fragments within the WGS data [23]. Sequence type (ST) identification was done with the mlst software (Seemann T, https://github.com/tseemann/mlst) using MLST schemes from the PubMLST website (https://pubmlst.org/) [24].

Core genomes were aligned using parsnp tool from Harvest suite v1.1.2 [25]. Thereafter, recombinations in the core genomes were detected using BratNextGen software [26]. For phylogenetic analysis, recombination-free alignments were created by masking all significant recombinant segments as missing data in the input alignment. These alignments were used to reconstruct a maximum likelihood phylogenetic tree with RaxML using the GTR-GAMMA model [27].

As core genome alignment and MLST analysis resulted in similar clustering, the data are presented according to STs of MLST.

### 2.7. Testing for Resistance Genes

Beta-lactamases were detected using a homology search against the ResFinder database. Contigs containing ESBL-genes were further analyzed using BLAST [28] against the NCBI nt/nr databases to specify possible relation to known plasmid sequences and to check for the presence of mobile elements near the ESBL-genes.

### 2.8. Statistical Analysis

Statistical analysis used Stata 14.2 [29]. As a pilot study, the sample size of volunteers was not formally calculated. Descriptive statistics are presented as a comparison of ESBL-Enterobacteriaceae carriers and non-carriers. Following exploratory variables were used in univariate analysis: age, gender, occupation, the main source of milk products, fruit and vegetables, meat and drinking water; owning domestic animals, owning a dog, owning a cat, owning farm animals, travelling abroad, travelling abroad to Europe, travelling outside Europe, smoking, eating outside the home, antibiotic treatment and hospitalization in previous year. Factors that were statistically significant in univariate analysis (*p* < 0.05) were included in multiple logistic regression analysis to determine independent risk factors associated with the colonization of ESBL-producing strain. As variance inflation factor (VIF = 10.81) indicated multicollinearity between travelling in Europe and travelling in general, the travelling in Europe was excluded from multiple regression analysis. The bottled water category was excluded from further analysis as it consisted of only 5 participants (2%). Likelihood ratio test and Akaike information criterion were used to determine by backward selection the best final model. The final model included occupation and travelling as independent variables. A Chi square goodness of fit test was used, for final model Chi(2) = 2.94, *p* = 0.230.

Availability of data and materials: The datasets used and/or analyzed during the current study available from the corresponding author on reasonable request. All WGS data have been deposited into the NCBI (PRJNA311519; Accession numbers: JABUFH000000000, JABUFI000000000, JABUFJ000000000, JABUFK000000000, JABUFL000000000, JABUFM000000000, JABUFN000000000, JABUFO000000000, JABUFP000000000, JABUFQ000000000, JABUFR000000000, JABUFS000000000, JABUFT000000000, JABUFU000000000, JABUFV000000000, JABUFW000000000, JABUFX000000000, JABUFY000000000, JABUFZ000000000, JABUGA000000000, JABUGB000000000, JABUGC000000000, JABUGD000000000).

## 3. Results

### 3.1. Study Population

We contacted 200 veterinarians and 63 pig farms of which 29 (14.5%) and nine (14.3%), respectively agreed to participate. A total of 207 individuals were recruited—29 veterinarians, 29 pig farmers (one to five per farm), 80 dog owners and 69 individuals in the control group.

Majority of the subjects were female (*n* = 144; 70%), with a median age of 40 years (range 6 months–82 years); 46 (22%) were aged <18 years.

Colonization with ESBL-Enterobacteriaceae occurred only in fecal samples. Altogether 14 (6.8%; 95%CI: 3.75–11.09%) individuals were colonized, namely seven (24.1%; 95% CI: 10.3–43.54%) pig farmers, two (6.9%; 95% CI 0.84–22.77%) veterinarians, one (1.3%; 95% CI: 0.03–6.7%) dog owner, and four (5.8%; 95% CI: 1.60–14.18%) in the control group. The only isolated ESBL-Enterobacteriaceae species was *E. coli*.

### 3.2. Risk Factors of ESBL-Enterobacteriacae Carriage

Comparison of ESBL-Enterobacteriaceae positive and negative subjects is presented in Table 1.

According to univariate analysis, occupation as a pig farmer, consumption of self- or local farmer produced vegetables, drinking bottled water, and eating mainly at home were associated with ESBL-Enterobacteriaceae carriage. Traveling in Europe turned out as a protective factor. All statistically significant variables from univariate analysis were included in multiple regression analysis to exclude possible cofounders.

The multiple logistic regression model showed that the only independent predictor of colonization with ESBL-Enterobacteriaceae was occupation as a pig farmer compared to the “other/not working” group (OR 4.8; 95%CI 1.2–19.1).

### 3.3. Phylogenetic Grouping, Antimicrobial Susceptibility and ESBL Encoding Genes

In total, 23 ESBL-Enterobacteriaceae strains were obtained. We aimed to isolate at least two strains from each participant, but this was not feasible in five cases due to poor growth of the isolates.

**Figure 1:**
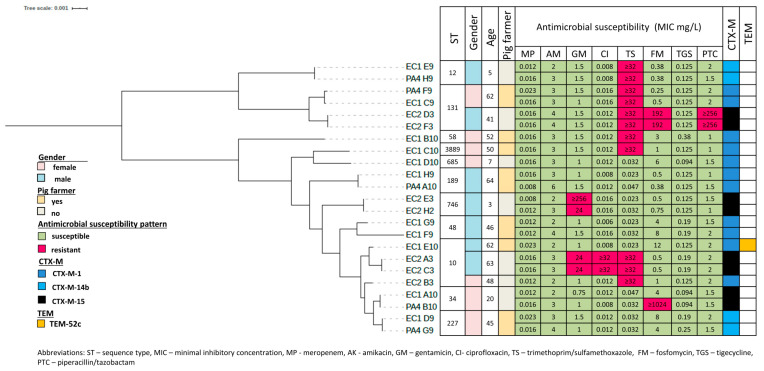
Main characteristics of ESBL-Enterobacteriaceae hosts (*n* = 14) and strains (*n* = 23). A maximum likelihood core genome tree and MLST analysis; presence of ESBL genes. Tested antimicrobial susceptibilities are presented as follows: green color—susceptible and red—resistant strain.

All strains isolated from the selective medium showed positive ESBL production in confirmatory E-tests. Four of the 23 ESBL producing strains were resistant to three different antibiotic groups—non-susceptibility to trimethoprim-sulfamethoxazole being the most common (11 out of 23 strains; Figure 1).

As all MIC values to meropenem were under 0.125 mg/L, carbapenemase production was not tested [30].

In MLST analysis, the strains distributed into 11 sequence types (ST). Only two STs were identified in more than one person (both ST10 and ST131, with two strains from two individuals each) (Figure 1).

**Figure 2:**
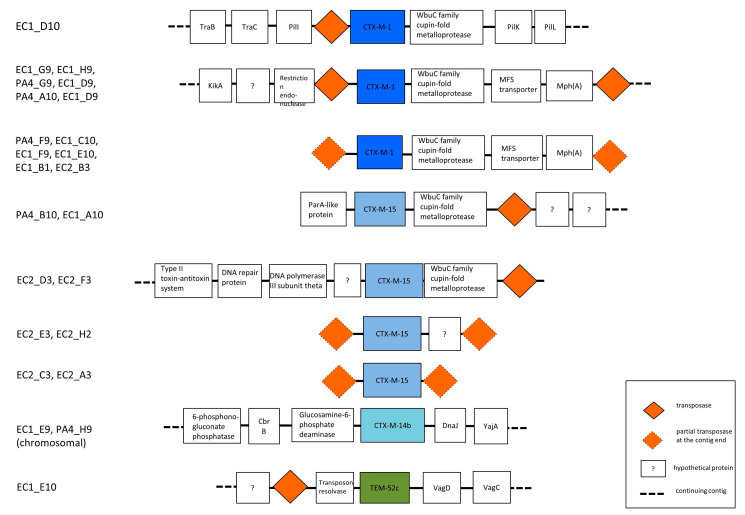
The surrounding context of ESBL-genes in studied strains. Comparison to available annotated plasmid sequences in public databases showed that most of the corresponding contigs contained mobile elements and plasmid-related genes. Figure illustrates only the context and is not scaled to the actual length of the genes and intergenic sequences.

As shown in Figure 1, *bla*_CTX-M_ genes were isolated in all ESBL-Enterobacteriaceae strains, whereas only one strain carried *bla*_TEM_ gene. Three types of CTX-M class genes were identified. *Bla*_CTX-M-1_ (13 strains from 9 individuals) was the most common followed by *bla*_CTX-M-15_ (8 strains from 4 individuals) and *bla*_CTX-M-14_ (2 strains from 1 individual). *Bla*_CTX-M-1_ producing Enterobacteriaceae were more frequently found in pig farmers than in other populations (7/29; 24.1% vs. 2/178; 1.1%) while *bla*_CTX-M-14_ and *bla*_CTX-M-15_ only occurred in other populations and not in pig farmers. Most of the ESBL gene containing contigs had mobile elements in the vicinity of *bla*-genes (21/23 *bla*_CTX-M_, Figure 2) and contained several other plasmid related genes suggesting possible plasmid origin. No carbapenemase encoding genes were found.

## 4. Discussion

In the first study conducted in Estonia looking at the colonization of ESBL-Enterobacteriaceae in the population, of whom majority had contact with pets and/or farm animals, we found: (1) that the carriage of ESBL-Enterobacteriaceae was relatively low in most groups except pig farmers; (2) accordingly the only independent risk factor for ESBL-Enterobacteriaceae colonization was working as a pig farmer; (3) all ESBL-positive isolates were *E. coli*; (4) the most prevalent ESBL gene was *bla*_CTX-M-1_, isolated predominantly from pig farmers; (5) the spread of ESBL-Enterobacteriaceae was allodemic rather than epidemic or endemic, and (6) the co-resistance rate among ESBL-Enterobacteriaceae strains was low, except to TMP/SMX.

The reported prevalence of ESBL-Enterobacteriaceae, varies significantly across the world, ranging between 1.6% in Latvia to 70% in some provinces of China and Thailand [3,7,31,32]. Geographic variances might have developed mainly due to different antibiotic policies and hygiene standards in both human medicine and animal husbandry, but also of the level of access to basic sanitation and clean water. The latter contributes to the formation and transmission of resistance reservoirs between environments [6]. Our finding of 6.8% of fecal carriage of ESBL-Enterobacteriaceae in total is in concordance with reports from other Northern European countries with rates between 4.7 and 6.6% [3,33,34]. This result was fairly predictable considering Estonia’s good sanitation level and prudent antibiotic consumption strategies in human medicine [35].

Although overall antibiotic usage in veterinary medicine is low, the proportion of 3rd and 4th generation cephalosporins, however, is exceptionally high. Estonia holds the first place in Europe on this. The latter is of concern as extensive use of 3rd and 4th generation cephalosporins in animal husbandry has been suggested to increase the development and transmission of *bla*_CTX-M_ genes from farm animals (poultry, pigs, cattle) to the human population [6].

Our findings of significantly higher colonization rate of ESBL-Enterobacteriaceae among pig farmers (24.1%) than in any the other groups (3.9%), including dog owners and veterinarians support previous studies indicating that contact to pigs is a risk factor for acquiring ESBL-Enterobacteriaceae [36]. This prevalence is much higher than in other European countries such as the Netherlands, Germany and Denmark, in which the carriage rates of farm workers have been similar to the general population (2.5 to 13%) [36,37,38,39]. Rates similar to ours have been reported in China (20%) where colonization with ESBL-Enterobacteriaceae in healthy non-animal exposed humans was also very high, reaching up to 70% and carriage rate in pigs is reported above 60% [4,40]. In contrast to farmers, the phenotypic resistance to 3rd generation cephalosporins isolated from pigs in Estonia has been reported as low, i.e., 2.5% and 3.3% in healthy and 4.2% and 7.7% in diseased animals, for cefotaxime and ceftazidime, respectively [41]. Interestingly no ESBL-*Enterobacteriaceae* strains were identified from animals or environment of the farms where workers with ESBL-*Enterobacteriaceae* carriage were detected (unpublished preliminary data).

As the transmission of methicillin-resistant staphylococci from pigs to humans has been well described, evidence of a role of contact with pigs in human ESBL-Enterobacteriaceae colonization is still limited. Direct contact with pigs is assumed to be the main transmission route, and carriage rate depends on the contact intensity and pig ESBL-Enterobacteriaceae status, but further discussions have led to a possible connection with farm air contamination and inhalation of resistant enterobacteria or their genetic elements by workers, followed by carriage in the nostrils [37,38,42,43]. Similar to Fischer et al., we did not find any ESBL-Enterobacteriaceae in volunteers’ nares, making this hypothesis not well justified.

The predominance of *bla*_CTX-M-1_ is an unusual finding in the community-based studies, as *bla*_CTX-M-15_ dominates in human studies in other Nordic countries and Germany and *bla*_CTX-M-14_ in Mexican, Portuguese and Asian volunteers [5,6,10,32,34,44,45,46]. *Bla*_CTX-M-1_ is the most frequent ESBL-gene in livestock, and transmission of this lineage from animals through food or via direct contact with farm animals (including pigs) has been described [6,37,38,43].

The role of *bla*_CTX-M-1_ in causing human disease remains unclear as it is rarely found in clinically relevant samples of hospitalized patients in Estonia or elsewhere [47,48]. Even if *bla*_CTX-M-1_ carrying isolates have been found as clinically relevant, it has occurred in Germany and the Netherlands, both countries having large-scale intensive farming [6,14].

We found an allodemic spread pattern rather than a single-clone epidemic as 11 different sequence types of ESBL-producing *E. coli* was carried by 14 volunteers. Similar to previous studies in healthy volunteers, there was no dominance of ST131, indicating that each carrier has its strain and that ESBL transfer from one person to another in the non-closed community is unlikely, with only genetic fragments (like beta-lactamase coding genes) being transmitted [7,10].

As blaCTX-M containing plasmids often involve cassettes encoding resistance to multiple antibiotic classes, the multiresistant phenotype is typical in these strains [1,6]. We did not observe multi-resistance, but almost half of the isolates (47.8%) were resistant to TMP/SMX, similar to a previous multi-country study of Ny et al. (2018). At the same time, the most frequently described co-resistance in ESBL-producing isolates, resistance to ciprofloxacin, from 10% in Norwegian to 65% in Russian volunteers, was at the lower end in our strains (8.7%) [3,34].

A few limitations should be noted. We must emphasize first that we specifically recruited groups of individuals who had contact with animals. Second, we excluded patients with a history of hospitalization and antibiotic treatment in the previous three months, the latter being a well-known risk factor for the development and spread of antimicrobial resistance. Thus, the preselected group may not necessarily be fully representative of the general Estonian population. As this was a pilot study, no formal sample size calculation was performed. Still, we believe that the number of subjects should be sufficient to draw preliminary conclusions and suggestions for further studies.

## 5. Conclusions

The prevalence of ESBL-Enterobacteriaceae in the Estonian community is generally low except among pig farmers. Working as a pig farmer is an independent risk factor for the acquisition of ESBL-Enterobacteriaceae, suggesting a possible transfer of antibiotic-resistant strains from pigs to humans. The clinical relevance of this transfer, however, is less clear as the majority of human infections have been related to CTX-M-14 or CTX-M-15 genotypes, and not to the CTX-M-1 genotype that was found in pig farmers in our study.

## Figures and Tables

**Table 1:** Association between carriage of ESBL-Enterobacteriaceae and risk factors/clinical characteristics.

	ESBL-Enterobacteriaceae Carriage		Univariate Analysis	
	Negative (*n* = 193)	Positive (*n* = 14)	OR (95%CI)	*p*-Value
Male sex (%)	56 (29)	7 (50)	2.4 (0.8–7.3)	0.109
Age: median (range)	40 (0–82)	47 (3–64)	NA	
Occupation (%) Other/not working ^a^ Veterinarian Pig farmer	141 (73.1) 30 (15.5) 22 (11.4)	5 (35.7) 2 (14) 7 (50)	1 1.9 (0.3–10.1) 9.0 (2.6–30.8)	0.463 <0.001 *
Source of food ^b^				
Milk products (%) Purchased from market/shop Own produce/local farmer Meat (%) Purchased from market/shop Own produce/local farmer Fruit and vegetables Purchased from market/shop Own produce/local farmer	154 (82.4) 33 (17.7) 156 (80.8) 34 (17.9) 93 (48.2) 98 (51.3)	10 (71.4) 4 (28.6) 11 (78.6) 3 (21.4) 2 (14.3) 12 (85.8)	1 1.9 (0.5–6.3) 1 1.2 (0.3–4.7) 1 5.7 (1.2–26.1)	0.316 0.741 0.025 *
Source of the drinking water (%) Central water supply Well Bottled water Various sources	128 (67.0) 55 (28.8) 3 (1.6) 5 (2.6)	6 (42.8) 6 (42.9) 2 (14.3) 0 (0)	1 2.3 (0.7–7.5) 14.2 (2.0–101.7) NA	0.159 0.008 *
Smoking (%)	21 (10.9)	4 (28.6)	3.3 (0.9–11.3)	0.063
Owning domestic animals (%) Dog Cat Farm animals ^c^	150 (77.7) 126 (65.3) 92 (47.7) 21 (10.9)	10 (71.4) 8 (57.1) 8 (57.1) 2 (14.3)	0.7 (0.2–2.4) 0.7 (0.2–2.1) 1.5 (0.5–4.3) 1.3 (0.3–6.5)	0.589 0.539 0.495 0.697
Travel abroad in past 12 months (%) Europe Outside Europe	110 (57) 105 (54.4) 17 (8.8)	3 (21.4) 3 (21.4) 2 (14.3)	0.2 (0.1–0.8) 0.2 (0.1–0.8) 1.7 (0.4–8.4)	0.018 * 0.023 * 0.498
Eating outside home (%) ^d^ Few times (<3 times) a year/never Frequently (≥3 times a month)	90 (46.9) 102 (53.1)	11 (78.6) 3 (21.4)	1 0.2 (0.1–0.9)	0.033 *
Antibacterial treatment in previous year (%)	53 (27.5)	2 (14.3)	2.3 (0.5–10.5)	0.293
Hospitalization in previous year (%)	21 (11.1)	0 (0)	NA	

* Statistically significant (*p* < 0.05). ^a^ Occupational data were initially assigned to five different categories – healthcare worker, veterinary personal, (pig) farmer, food industry worker (exposure to raw meat, unpasteurized milk or unprocessed eggs or both), and another status including the unemployed. As some groups (healthcare workers, food industry workers) were small (seven and three persons, respectively), they were combined with other/unemployed group in further analysis. ^b^ Subjects who reported that they do not consume this type of food were removed from analysis. Accordingly 6 people were removed from milk product, 3 people from meat and 2 people from fruit and vegetable source analysis.^c^ Cattle, swine ^d^ 1 person who did not answer the question was excluded from analysis.
